# Rapid detection of MERS coronavirus-like viruses in bats: potential for tracking MERS coronavirus transmission and animal origin

**DOI:** 10.1038/s41426-017-0016-7

**Published:** 2018-03-07

**Authors:** Patrick C. Y. Woo, Susanna K. P. Lau, Yixin Chen, Emily Y. M. Wong, Kwok-Hung Chan, Honglin Chen, Libiao Zhang, Ningshao Xia, Kwok-Yung Yuen

**Affiliations:** 1 State Key Laboratory of Emerging Infectious Diseases, The University of Hong Kong, Hong Kong, China; 2 Research Centre of Infection and Immunology, The University of Hong Kong, Hong Kong, China; 3 Collaborative Innovation Center for Diagnosis and Treatment of Infectious Diseases, The University of Hong Kong, Hong Kong, China; 4 Department of Microbiology, The University of Hong Kong, Hong Kong, China; 5 State Key Laboratory of Molecular Vaccinology and Molecular Diagnostics & National Institute of Diagnostics and Vaccine Development in Infectious Diseases, School of Life Sciences, School of Public Health, Xiamen University, Xiamen, 361102 Fujian, China; 6 Guangdong Key Laboratory of Animal Conservation and Resource Utilization, Guangdong Public Laboratory of Wild Animal Conservation and Utilization, Guangdong Institute of Applied Biological Resources, Guangzhou, China

## Abstract

Recently, we developed a monoclonal antibody-based rapid nucleocapsid protein detection assay for diagnosis of MERS coronavirus (MERS-CoV) in humans and dromedary camels. In this study, we examined the usefulness of this assay to detect other lineage C betacoronaviruses closely related to MERS-CoV in bats. The rapid MERS-CoV nucleocapsid protein detection assay was tested positive in 24 (88.9%) of 27 *Tylonycteris* bat CoV HKU4 (Ty-BatCoV-HKU4) RNA-positive alimentary samples of *Tylonycteris pachypus* and 4 (19.0%) of 21 *Pipistrellus* bat CoV HKU5 (Pi-BatCoV-HKU5) RNA-positive alimentary samples of *Pipistrellus abramus*. There was significantly more Ty-BatCoV-HKU4 RNA-positive alimentary samples than Pi-BatCoV-HKU5 RNA-positive alimentary samples that were tested positive by the rapid MERS-CoV nucleocapsid protein detection assay (*P* < 0.001 by Chi-square test). The rapid assay was tested negative in all 51 alimentary samples RNA-positive for alphacoronaviruses (*Rhinolophus* bat CoV HKU2, *Myotis* bat CoV HKU6, *Miniopterus* bat CoV HKU8 and *Hipposideros* batCoV HKU10) and 32 alimentary samples positive for lineage B (SARS-related *Rhinolophus* bat CoV HKU3) and lineage D (*Rousettus* bat CoV HKU9) betacoronaviruses. No significant difference was observed between the viral loads of Ty-BatCoV-HKU4/Pi-BatCoV-HKU5 RNA-positive alimentary samples that were tested positive and negative by the rapid test (Mann-Witney *U* test). The rapid MERS-CoV nucleocapsid protein detection assay is able to rapidly detect lineage C betacoronaviruses in bats. It detected significantly more Ty-BatCoV-HKU4 than Pi-BatCoV-HKU5 because MERS-CoV is more closely related to Ty-BatCoV-HKU4 than Pi-BatCoV-HKU5. This assay will facilitate rapid on-site mass screening of animal samples for ancestors of MERS-CoV and tracking transmission in the related bat species.

## Introduction

Since its first appearance in 2012, the Middle East Respiratory Syndrome (MERS) has affected more than 25 countries in four continents with more than 1300 cases and a high fatality rate of more than 30% ^1^. A novel lineage C betacoronavirus, MERS coronavirus (MERS-CoV), has been confirmed to be the etiological agent^2,3^. Human dipeptidyl peptidase 4 (hDPP4) was found to be the cellular receptor for MERS-CoV^4^. Subsequent detection of MERS-CoV and its antibodies in dromedaries in various countries in the Middle East and North Africa have implied that these animals are probably the reservoir for MERS-CoV^5–7^. Other lineage C betacoronaviruses in bats [e.g., *Tylonycteris* bat CoV HKU4 (Ty-BatCoV-HKU4), *Pipistrellus* bat CoV HKU5 (Pi-BatCoV-HKU5)] and hedgehogs were found to be closely related to MERS-CoV^8,9^.

So far, detection of MERS-CoV and discoveries of its closely related CoVs are most efficiently achieved through RT-PCR. Although RT-PCR is highly sensitive, its turn-around-time is about 4 h and the test requires expensive equipment, stringent laboratory set-up and personal attention to prevent laboratory PCR product cross contamination which may lead to false-positive results. Recently, we have developed a monoclonal antibody-based rapid nucleocapsid protein detection assay for on-site diagnosis of MERS-CoV, which can be finished in 30 min^10^. This rapid test is highly specific for MERS-CoV for human and dromedary samples, as samples containing other human CoVs (HCoV-OC43, HCoV-229E, HCoV-NL63, and HCoV-HKU1) or dromedary CoV UAE-HKU23 all showed negative results. However, these human and dromedary CoVs are all non-lineage C betacoronaviruses. It is not known if the nucleocapsid protein of lineage C betacoronaviruses other than MERS-CoV may cross-react with that of MERS-CoV. We hypothesize that such cross-reactivity may occur and the rapid test can pick up lineage C betacoronaviruses closely related to MERS-CoV; and hence would be useful for the discovery of MERS-CoV ancestors. To test this hypothesis, we examined the usefulness of this rapid test to detect four alphacoronaviruses and four lineage B, C and D betacoronaviruses that we previously discovered in alimentary samples of bats^8,11–16^. The differential positive rate of detecting lineage C betacoronaviruses that showed different relatedness to MERS-CoV was also discussed.

## Materials and methods

### Samples

A total of 131 alimentary samples from bats that were tested RNA-positive for four alphacoronaviruses (*Rhinolophus* bat CoV HKU2, *Myotis* bat CoV HKU6, *Miniopterus* bat CoV HKU8 and *Hipposideros* batCoV HKU10) and four lineage B (SARS-related *Rhinolophus* bat CoV HKU3), C (Ty-BatCoV-HKU4 and Pi-BatCoV-HKU5) and D (*Rousettus* bat CoV HKU9) betacoronaviruses collected in Hong Kong and the Guizhou Province, China, over a 9-year period (2006 to 2015) were included in the study (Table 1)^8,11–16^. In addition, serial dilutions (2.4, 1.2, 0.6, and 0.3 μg) of the purified recombinant nucleocapsid protein of a Ty-BatCoV-HKU4 strain (SM2A) of which the sample showed positive rapid test result, three Ty-BatCoV-HKU4 RNA-negative alimentary samples of *Tylonycteris pachypus* and three Pi-BatCoV-HKU5 RNA-negative alimentary samples of *Pipistrellus abramus* were also included as controls.

**Table 1:** Alimentary samples tested in the present study

Bat	CoV found positive	Number of samples	Number (%) of samples tested positive by rapid MERS-CoV nucleocapsid protein detection assay
*Rhinolophus sinicus*	Rh-BatCoV-HKU2	16	0
*Rhinolophus sinicus*	SARSr-Rs-BatCoV-HKU3	23	0
*Tylonycteris pachypus*	Ty-BatCoV-HKU4	27	24 (88.9%)
*Pipistrellus abramus*	Pi-BatCoV-HKU5	21	4 (19.0%)
*Myotis ricketti*	My-BatCoV-HKU6	7	0
*Miniopterus pusillus*	Mi-BatCoV-HKU8	8	0
*Rousettus leschenaultii*	Ro-BatCoV-HKU9	9	0
*Hipposideros pomona*	Hi-BatCoV-HKU10	20	0

### Cloning and purification of (His)_6_-tagged recombinant nucleocapsid protein of Ty-BatCoV-HKU4

Cloning and purification of the (His)_6_-tagged recombinant nucleocapsid protein of Ty-BatCoV-HKU4 was performed using a protocol we described previously^17,18^. To produce a plasmid for protein purification, primers (5′-ACT CGC TAG CAT GGC AAC TCC TGC TGCA CCT-3′ and 5′-TCA TGC TAG CTC AAG CCT CTG AAT CGA AGG TAA-3′) were used to amplify the gene encoding the nucleocapsid protein of Ty-BatCoV-HKU4 strain SM2A by RT-PCR. The sequence coding for amino acid residues 1 to 423 of the nucleocapsid protein was amplified and cloned into the *Nhe*I site of expression vector pET-28b(+) (Novagen, Merck Millipore, Billerica, MA, USA) in frame and upstream of the series of six histidine residues. The recombinant nucleocapsid protein was expressed and purified using Ni-NTA affinity chromatograph column (Qiagen, Hilden, Germany) according to the manufacturer’s instructions.

### Rapid MERS-CoV nucleocapsid protein detection assay

The rapid MERS-CoV nucleocapsid protein detection assay, in the format of a lateral flow immunoassay, was performed as described previously^10^, using LFIA test strip (Millipore, Bedford, MA, USA) and two highly specific monoclonal antibodies against the MERS-CoV recombinant nucleocapsid protein (MD3E9 for coating and MD8B6 for conjugating). Testing was carried out by mixing 380 µl of each sample (originally collected by mixing a fecal swab with 1 ml virus transport medium) with 20 µl of lysis buffer for 10 s at room temperature. Then, 80 µl of the pre-treated sample was transferred by pipette into the sample well of the test card slowly at room temperature. The results are read visually in 30 min. The result is considered negative when the control line is positive but the test line negative and considered positive when both the control and test lines are positive. If the control line is negative, the test is considered invalid.

### Quantitative real-time RT-PCR

Viral loads of Ty-BatCoV-HKU4 and Pi-BatCoV-HKU5 were performed by quantitative real-time RT-PCR as described previously^10,19^. RNA was amplified in a LightCycler instrument with SuperScript III Platinum One-Step Quantitative RT-PCR System (Invitrogen, San Diego, CA, USA) using forward primer 5′-CGG AAA ATC AAC ACC GGT AAT GGT-3′, reverse primer 5′-TAG CCT CTG GTC CAG TCC CA-3′ and probe 5′-(FAM)-TTA AAC AAT TGG CYC CCA GAT GGT TCT TCT ACT ACA-(BHQ-1)-3′ for Ty-BatCoV-HKU4 and forward primer 5′-CCC AGA CCT GCC CCT AAT AC-3′, reverse primer 5′-AAG AGG CTG CTT ACC GTG CT-3′ and probe 5′-(FAM)-CAC TGT CTC ATG GTT CAC GGG CCT TAC-(IBFQ)-3′ for Pi-BatCoV-HKU5. For quantitative analysis, a reference standard was prepared using the pCRII-TOPO vector (Invitrogen, San Diego, CA, USA) containing the target sequence. Calibration curve was generated by serial 10-fold dilutions equivalent to 10^2^–10^9^ copies per reaction mixture parallel with test samples.

### Phylogenetic analysis

Phylogenetic tree was constructed using N gene with the maximum likelihood method based on substitution model of Whelan and Goldman model with gamma distribution (WAG+G) by MEGA 6.0. The best-fit substitution model was selected using MEGA 6.0.

## Results

### Detection of lineage C betacoronaviruses by rapid MERS-CoV nucleocapsid protein detection assay

**Fig. 1:**
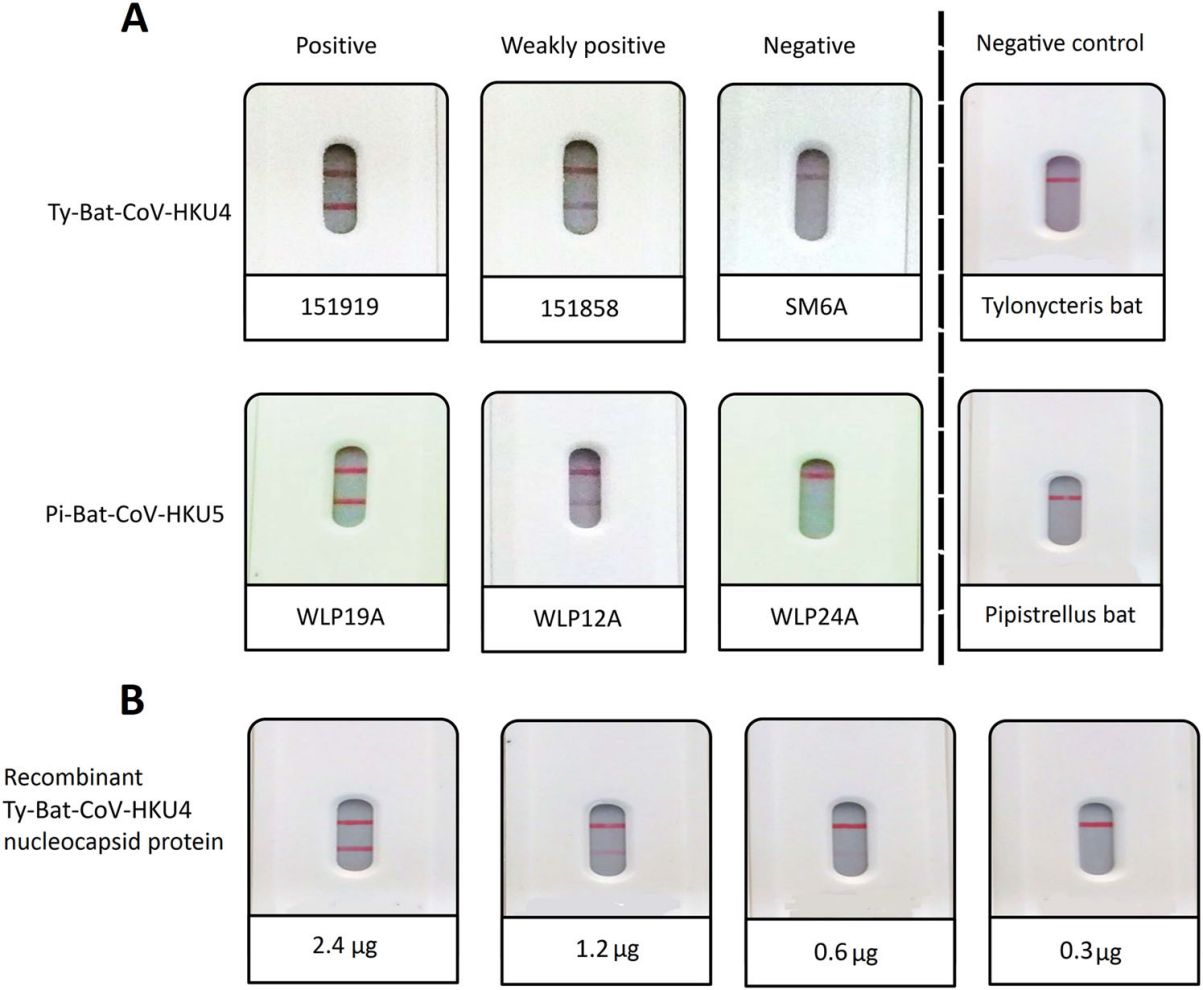
Rapid MERS-CoV nucleocapsid protein detection assay showing. **a** positive, weakly positive and negative results for selected Ty-BatCoV-HKU4 positive and Pi-BatCoV-HKU5 positive samples and negative results for *Tylonycteris pachypus* and *Pipistrellus abramus* CoV RNA-negative samples; and (**b**) results for serial dilutions of recombinant nucleocapsid protein of Ty-BatCoV-HKU4 strain SM2A

The rapid MERS-CoV nucleocapsid protein detection assay was tested positive in 24 (88.9%) of the 27 Ty-BatCoV-HKU4 RNA-positive alimentary samples of *Tylonycteris pachypus* and 4 (19.0%) of the 21 Pi-BatCoV-HKU5 RNA-positive alimentary samples of *Pipistrellus abramus* (Fig 1a and Table 1). There was significantly more Ty-BatCoV-HKU4 RNA-positive alimentary samples than Pi-BatCoV-HKU5 RNA-positive alimentary samples that were tested positive by the rapid MERS-CoV nucleocapsid protein detection assay (*P* < 0.001 by Chi-square test). The rapid MERS-CoV nucleocapsid protein detection assay was tested negative in all 51 alimentary samples RNA-positive for alphacoronaviruses and 32 alimentary samples RNA-positive for lineage B and lineage D betacoronaviruses (Table 1).

The purified recombinant nucleocapsid protein of a Ty-BatCoV-HKU4 strain (SM2A) of which the sample showed positive result was tested positive (Fig. 1b), and three Ty-BatCoV-HKU4 RNA-negative alimentary samples of *Tylonycteris pachypus* and three Pi-BatCoV-HKU5 RNA-negative alimentary samples of *Pipistrellus abramus* were tested negative (Fig. 1a), by the rapid test, confirming that the positive results were due to the nucleocapsid protein of the CoV, but not other ingredients or chemicals in the alimentary samples of the bats. For the recombinant nucleocapsid protein of Ty-BatCoV-HKU4 strain SM2A, the detection limit was 0.6 µg of protein.

### Quantitative real-time RT-PCR

The median (range) viral loads of the 27 Ty-BatCoV-HKU4 and 21 Pi-BatCoV-HKU5 positive alimentary samples were 4.08 × 10^8^ (8.91 × 10^6^–6.31 × 10^10^) copies/g and 7.01 × 10^6^ (1.24 × 10^5^–2.68 × 10^8^) copies/g respectively (Table 2). No significant difference was observed between the viral loads of Ty-BatCoV-HKU4/Pi-BatCoV-HKU5 RNA-positive alimentary samples that were tested positive and negative by the rapid MERS-CoV nucleocapsid protein detection assay (Mann–Witney *U* test).

**Table 2:** Rapid test results and viral loads of Ty-BatCoV-HKU4 and Pi-BatCoV-HKU5 positive samples

Sample number	Year of collection	Site of collection	Viral load (copy number/g)	Rapid test result
Ty-BatCoV-HKU4 positive samples				
SM1A	2010	Hong Kong	5.78 × 10^8^	+
SM2A	2010	Hong Kong	5.46 × 10^8^	w+
SM3A	2010	Hong Kong	8.91 × 10^6^	w+
SM5A	2010	Hong Kong	6.31 × 10^10^	+
SM6A	2010	Hong Kong	2.01 × 10^9^	−
SM7A	2010	Hong Kong	6.82 × 10^8^	+
SM9A	2010	Hong Kong	3.67 × 10^7^	−
SM12A	2010	Hong Kong	1.83 × 10^8^	w+
SM13A	2010	Hong Kong	9.32 × 10^6^	−
151707	2015	Guizhou	2.90 × 10^7^	w+
151708	2015	Guizhou	7.60 × 10^8^	+
151710	2015	Guizhou	9.94 × 10^9^	+
151823	2015	Guizhou	1.23 × 10^8^	w+
151824	2015	Guizhou	4.17 × 10^7^	+
151829	2015	Guizhou	2.87 × 10^8^	+
151830	2015	Guizhou	2.56 × 10^7^	w+
151832	2015	Guizhou	1.31 × 10^9^	+
151857	2015	Guizhou	2.81 × 10^9^	+
151858	2015	Guizhou	2.53 × 10^8^	w+
151859	2015	Guizhou	4.09 × 10^7^	w+
151861	2015	Guizhou	2.28 × 10^9^	+
151862	2015	Guizhou	4.08 × 10^8^	+
151863	2015	Guizhou	1.48 × 10^8^	w+
151864	2015	Guizhou	1.71 × 10^8^	w+
151907	2015	Guizhou	1.13 × 10^9^	+
151912	2015	Guizhou	1.53 × 10^9^	+
151919	2015	Guizhou	6.78 × 10^8^	+
Pi-BatCoV-HKU5 positive samples				
WLP5A	2009	Hong Kong	2.45 × 10^7^	−
WLP11A	2009	Hong Kong	9.35 × 10^6^	−
WLP12A	2009	Hong Kong	1.10 × 10^8^	+
WLP16A	2009	Hong Kong	2.68 × 10^8^	−
WLP20A	2009	Hong Kong	7.01 × 10^6^	−
WLP02A	2009	Hong Kong	8.89 × 10^5^	−
WLP06A	2009	Hong Kong	1.24 × 10^5^	−
WLP10A	2009	Hong Kong	5.42 × 10^7^	−
WLP17A	2009	Hong Kong	2.79 × 10^7^	−
WLP19A	2009	Hong Kong	5.31 × 10^7^	+
WLP22A	2009	Hong Kong	2.59 × 10^6^	−
WLP23A	2009	Hong Kong	3.69 ×10^5^	−
WLP24A	2009	Hong Kong	8.15 × 10^6^	w+
WLP25A	2009	Hong Kong	1.01 × 10^6^	−
WLP26A	2009	Hong Kong	7.85 × 10^5^	−
WLP30A	2009	Hong Kong	1.71 × 10^6^	−
WLP31A	2009	Hong Kong	6.00 × 10^5^	w+
MP6A	2012	Hong Kong	9.29 × 10^6^	−
MP9A	2012	Hong Kong	8.93 × 10^6^	−
MP12A	2012	Hong Kong	3.54 × 10^6^	−
MP22A	2012	Hong Kong	3.34 × 10^6^	−

+ positive, w+ weakly positive, − negative

### Phylogenetic analysis

**Fig. 2:**
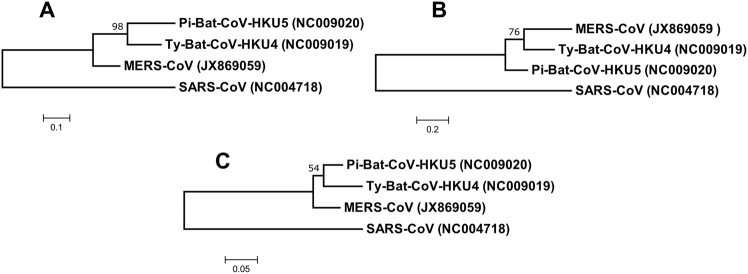
Phylogenetic analysis of (**a**) nucleocapsid, (**b**) spike and (**c**) RdRp protein of Ty-BatCoV-HKU4, Pi-BatCoV-HKU5, and MERS-CoV. The trees were constructed by maximum-likelihood method in MEGA 6 using substitution model WAG+G (nucleocapsid), WAG+F+I+G (spike) and LG+G (RdRp) with SARS-CoV as the outgroup. The percentage of trees in which the associated taxa clustered together next to the branches with bootstrap values was calculated from 1000 trees. The scale bars indicate the number of substitutions per 10, 5, and 20 amino acids, respectively

In the phylogenetic tree constructed using the amino acid sequences of the nucleocapsid protein of MERS-CoV, Ty-BatCoV-HKU4 and Pi-BatCoV-HKU5, it was observed that the nucleocapsid protein of MERS-CoV is more closely related to that of Ty-BatCoV-HKU4 than Pi-BatCoV-HKU5 (Fig. 2).

## Discussion

The rapid MERS-CoV nucleocapsid protein detection assay is able to rapidly detect lineage C betacoronaviruses in bats and other potential animal reservoir. Two years ago, we developed a monoclonal antibodies-based MERS-CoV nucleocapsid protein capture enzyme-linked immunosorbent assay using two MERS-CoV nucleocapsid protein specific monoclonal antibodies^20^. The two monoclonal antibodies reacted with the nucleocapsid protein of MERS-CoV but not those of HCoV-229E and HCoV-OC43^20^. Subsequently, using the format of a lateral flow immunoassay, we further developed a rapid MERS-CoV nucleocapsid protein detection assay^10^. Both the enzyme-linked immunosorbent assay and rapid assay showed high specificity for MERS-CoV in human and dromedary samples, as all the respiratory and alimentary samples that contained alphacoronaviruses and lineage A and B betacoronaviruses of human and dromedary origins, with comparable viral load ranges to those samples that contained Ty-BatCoV-HKU4 and Pi-BatCoV-HKU5 (data not shown), were tested negative by both assays^10,20^. In this study, we demonstrated and exploited the relative non-specificity of the rapid assay to detect Ty-BatCoV-HKU4 and Pi-BatCoV-HKU5, lineage C bat betacoronaviruses closely related to MERS-CoV.

**Fig. 3:**
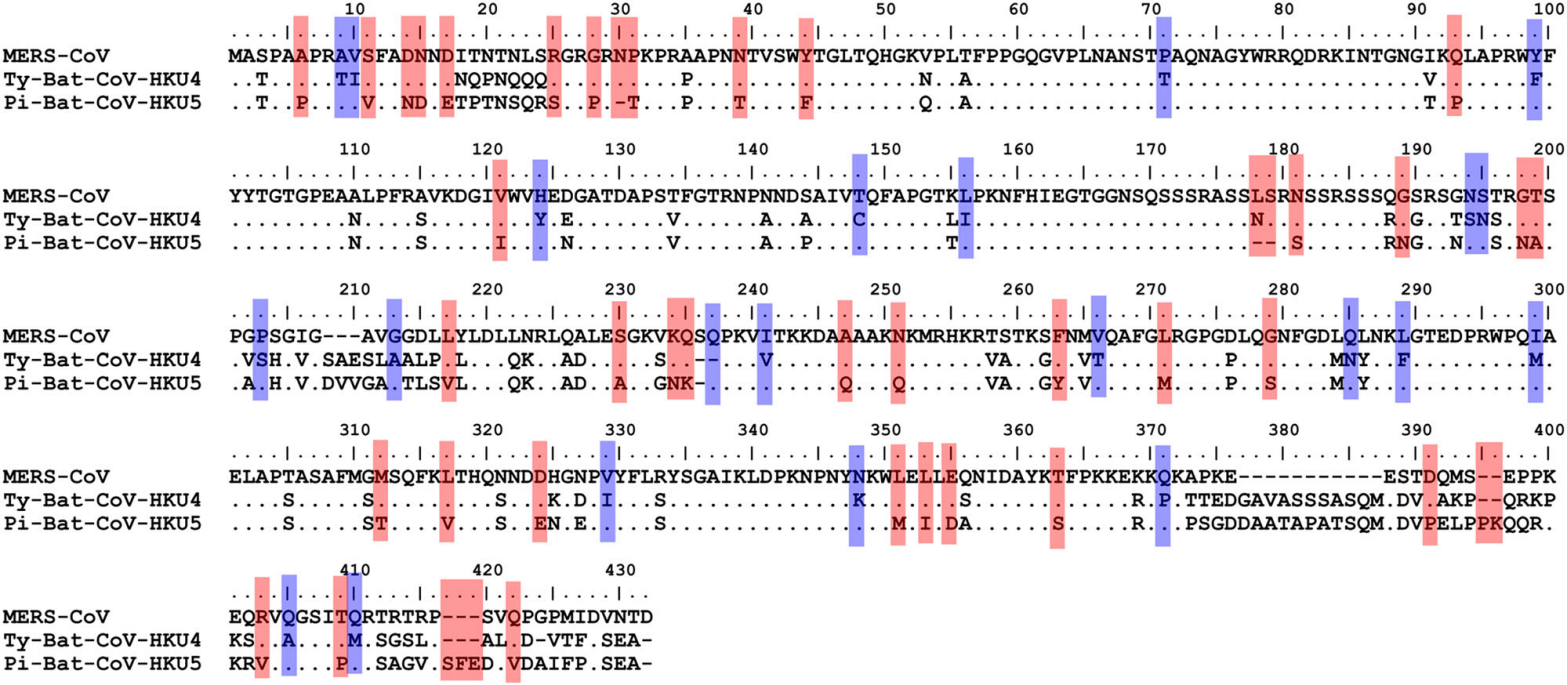
Multiple alignment of the amino acid sequences of the nucleocapsid proteins of MERS-CoV, Ty-BatCoV-HKU4, and Pi-BatCoV-HKU5. Red color shows the amino acid residues that were identical between the nucleocapsid proteins of MERS-CoV and Ty-BatCoV-HKU4 but not with that of Pi-BatCoV-HKU5. Blue color shows the amino acid residues that were identical between nucleocapsid proteins of MERS-CoV and Pi-BatCoV-HKU5 but not with that of Ty-BatCoV-HKU4

The rapid MERS-CoV nucleocapsid protein detection assay detected significantly more Ty-BatCoV-HKU4 than Pi-BatCoV-HKU5. Previous studies have shown that the receptor binding domain of the spike protein in Ty-BatCoV-HKU4 but not Pi-BatCoV-HKU5 is able to bind hDPP4^21,22^. Moreover, pseudotyped virus embedding the spike protein of Ty-BatCoV-HKU4 but not Pi-BatCoV-HKU5 can use hDPP4 as a receptor for infecting cells^21,22^. Furthermore, phylogenetic analysis showed that the S protein of MERS-CoV is more closely related to that of Ty-BatCoV-HKU4 than Pi-BatCoV-HKU5 (Fig. 2) and the receptor binding domain of Pi-BatCoV-HKU5 has deletions compared to those of MERS-CoV and Ty-BatCoV-HKU4^21^. As for the target nucleocapsid protein which the monoclonal antibodies of the rapid assay in the present study captured, phylogenetic analysis based on amino acid sequence comparison also showed that the nucleocapsid protein of MERS-CoV is more closely related to that of Ty-BatCoV-HKU4 than Pi-BatCoV-HKU5 (Fig. 2). This may explain why the rapid test is able to pick up almost 90% of Ty-BatCoV-HKU4, but only about 20% of Pi-BatCoV-HKU5, indicating that antigenically the epitopes on the nucleocapsid protein of Ty-BatCoV-HKU4 more readily cross-react with the corresponding ones on the nucleocapsid protein of MERS-CoV than Pi-BatCoV-HKU5. This is also in line with the results of multiple alignments for the amino acid sequences of the MERS-CoV, Ty-BatCoV-HKU4, and Pi-BatCoV-HKU5 nucleocapsid proteins. By comparing the variable sites of the three viruses, it was found that 44 residues were identical between MERS-CoV and Ty-BatCoV-HKU4 but not Pi-BatCoV-HKU5, while only 22 residues were identical between MERS-CoV and Pi-BatCoV-HKU5 but not Ty-BatCoV-HKU4 (Fig. 3). Based on the results, we speculate that whether a specific Ty-BatCoV-HKU4 or Pi-BatCoV-HKU5 strain can be picked up by the rapid test depends more on the sequence of the epitopes recognized by the monoclonal antibodies than the viral load of the sample, as high viral load samples (e.g., SM6A) could be tested negative while low viral load samples (e.g., WLP31A) could be tested positive (Table 2).

This rapid MERS-CoV nucleocapsid protein detection assay will facilitate rapid on-site mass screening of bat samples for ancestors of MERS-CoV that are closely related to MERS-CoV. So far, detection of MERS-CoV ancestors was performed by collection of animal samples, mostly from the Middle East and North Africa, transporting them to laboratories in developed countries, and viral RNA detection by quantitative real-time RT-PCR or metagenomics technologies^23,24^. Such viral studies were seldom performed in the field or laboratories in the Middle East where resources and expertize could be limited. Moreover, it is expensive and labor-intensive to perform these nucleic detection assays for screening thousands of bat samples from diverse species which may be needed to pick up just a dozen of positive samples in surveillance studies. Compared to quantitative real-time RT-PCR and metagenomics, the cost of the present rapid assay is much lower. It also does not require expertize on molecular technology and the expensive set-up for molecular studies, which may not be present in most clinical laboratories in the Middle East. Where specimen transport in hot climate is often a problem in the Middle East and North Africa, this user-friendly rapid test is suitable for testing a large number of bat samples at the study field. If a particular sample is positive, the investigator can easily collect more samples from the particular bat species for further studies. On the other hand, if a certain sample is negative, the bat can be released back to the wild. Such strategies would be extremely useful for tracking the transmission patterns of MERS-CoV and its close ancestors in different bat species across different countries. It is notable that although the rapid test is able to pick up 88.9% of Ty-BatCoV-HKU4 (a lineage C betacoronavirus which can bind and utilize hDPP4), it can only detect 19% of Pi-BatCoV-HKU5 (a lineage C betacoronavirus which cannot bind and utilize hDPP4). This limitation renders this rapid test mainly suitable for on-site mass screening for ancestors of MERS-CoV that are more closely related to MERS-CoV. Application of this rapid antigen assay for large number of samples at the field, coupled with confirmation by RT-PCR in more equipped laboratories, may speed up and allow more cost-effective surveillance studies for animal ancestors of MERS-CoV.
